# *De-novo* transcriptome assembly for gene identification, analysis, annotation, and molecular marker discovery in *Onobrychis viciifolia*

**DOI:** 10.1186/s12864-016-3083-6

**Published:** 2016-09-26

**Authors:** Marina Mora-Ortiz, Martin T. Swain, Martin J. Vickers, Matthew J. Hegarty, Rhys Kelly, Lydia M. J. Smith, Leif Skøt

**Affiliations:** 1 National Institute of Agricultural Botany, Huntingdon Road, Cambridge, CB3 OLE UK; 2 Aberystwyth University, IBERS, Gogerddan, Aberystwyth, Ceredigion SY23 3EB UK; 3 Present Address: School of Chemistry, Food Biosciences and Pharmacy, University of Reading, Whiteknights Campus, Reading, RG6 6AP UK; 4 Present Address: The Department of Cell and Developmental Biology, John Innes Centre, Norwich, NR4 7UH UK

**Keywords:** Transcriptome assembly, RNA-seq, *Onobrychis viciifolia*, Condensed tannins, Proanthocyanidins, SSR, Single nucleotide polymorphism

## Abstract

**Background:**

Sainfoin (*Onobrychis viciifolia*) is a highly nutritious tannin-containing forage legume. In the diet of ruminants sainfoin can have anti-parasitic effects and reduce methane emissions under in vitro conditions. Many of these benefits have been attributed to condensed tannins or proanthocyanidins in sainfoin. A combination of increased use of industrially produced nitrogen fertilizer, issues with establishment and productivity in the first year and more reliable alternatives, such as red clover led to a decline in the use of sainfoin since the middle of the last century. In recent years there has been a resurgence of interest in sainfoin due to its potential beneficial nutraceutical and environmental attributes. However, genomic resources are scarce, thus hampering progress in genetic analysis and improvement. To address this we have used next generation RNA sequencing technology to obtain the first transcriptome of sainfoin. We used the library to identify gene-based simple sequence repeats (SSRs) and potential single nucleotide polymorphisms (SNPs).

**Results:**

One genotype from each of five sainfoin accessions was sequenced. Paired-end (PE) sequences were generated from cDNA libraries of RNA extracted from 7 day old seedlings. A combined assembly of 92,772 transcripts was produced *de novo* using the Trinity programme. About 18,000 transcripts were annotated with at least one GO (gene ontology) term. A total of 63 transcripts were annotated as involved in the tannin biosynthesis pathway. We identified 3786 potential SSRs. SNPs were identified by mapping the reads of the individual assemblies against the combined assembly. After stringent filtering a total of 77,000 putative SNPs were identified. A phylogenetic analysis of single copy number genes showed that sainfoin was most closely related to red clover and *Medicago truncatula*, while *Lotus japonicus*, bean and soybean are more distant relatives.

**Conclusions:**

This work describes the first transcriptome assembly in sainfoin. The 92 K transcripts provide a rich source of SNP and SSR polymorphisms for future use in genetic studies of this crop. Annotation of genes involved in the condensed tannin biosynthesis pathway has provided the basis for further studies of the genetic control of this important trait in sainfoin.

**Electronic supplementary material:**

The online version of this article (doi:10.1186/s12864-016-3083-6) contains supplementary material, which is available to authorized users.

## Background

*Onobrychis viciifolia* or sainfoin is a perennial forage legume crop which contains condensed tannins or proanthocyanidins (PAs). Multiple benefits to animal nutrition and health have been attributed to the PA present in sainfoin. These benefits include anthelminthic properties, in vitro methane emission reduction in ruminants fed on this forage and prevention of the potentially life-threatening bloat associated with other non-PA producing forage legumes [1–5]. Sainfoin is also highly drought tolerant, due partly to its deep taproot and is resistant to most common pests and diseases. It also contributes to improving soil nitrogen levels due to atmospheric nitrogen fixation in root nodules by *rhizobia* [6, 7].

These benefits suggest that sainfoin could be an alternative to *Medicago sativa* (alfalfa) as a valuable forage crop. There are, however, a number of qualitative and agronomic issues that need to be addressed before this potential can be realised. Sainfoin has on average a 20 % lower yield than alfalfa. This is associated with poor establishment and a smaller leaf area. Also if the drill date is delayed until late spring, this normally prevents harvest in the first year. All these factors have discouraged growers from cultivating sainfoin more widely [8, 9] and its use has therefore declined. Another reason for its decline is the widespread use of inexpensive industrially produced nitrogen fertilizer. This has had a negative impact more generally on the use of forage legumes, not just sainfoin. This is compounded by the lack of systematic breeding or agronomic improvements in sainfoin. There is also a scarcity of basic genetic information available. The almost complete lack of molecular markers available has hampered the development of genetic diversity information in germplasm, as well as analysis of the genetic basis of complex traits from mapping families.

Next generation sequencing has revolutionized the potential for systematic crop genetic improvement, facilitating the study of genomes and transcriptomes [10–12]. RNA-seq can be used for gene identification, annotation, gene ontology, expression level analysis and SSRs and SNPs mining [13–15]. A significant advantage of this strategy is that it does not require previous knowledge of the genetic sequence of the organism. It is expected that RNA-seq will overtake other alternative methodologies for gene expression analysis due to the larger range of expression, base-pair resolution and higher sensitivity [16–18].

The primary aim of this work was to use next generation sequencing technology to develop molecular resources that will facilitate the development of genetic diversity analyses of germplasm and provide a platform for studying the genetic basis of PA biosynthesis in sainfoin. Sainfoin can be a diploid (2n = 2x = 14) or tetraploid (2n = 4x = 28) species; the former occurs rarely and is poorly characterized in the literature, whereas the latter can be considered representative of the majority of sainfoin accessions. Polyploidy has been associated with the domestication process of sainfoin in which more productive plants were selected [9, 19–21]. Both diploid and tetraploid accessions have a basic set of seven chromosomes [9, 20]. Tetraploid lines have been characterized as autopolyploids or allopolyploids. However, it is unclear whether the inheritance is tetrasomic or disomic in nature [19, 22–24]. A few EST-SSR (expressed sequence tag-simple sequence repeat) markers from *Medicago truncatula* have been validated in sainfoin, and some phylogenetic studies have been performed using sequence information from the Internal Transcribed Spacer Region (ITS) and matK markers [25]. Genomic and molecular resources in sainfoin are however, still under-developed [25–27]. To our knowledge, there are no molecular markers derived directly from sainfoin - nor have any *de novo* studies been conducted in this species.

Our knowledge of the content, structure and complexity of PAs in sainfoin germplasm is growing [28, 29], but little is known about the genetics of PA biosynthesis and its regulation. PAs are formed by polymerisation of flavan-3-ols, which in turn are products of a branch of the flavonoid biosynthesis pathway. The latter is well documented in many species [30, 31]. While a lot of progress has been made in recent years in *Arabidopsis thaliana* and forage legumes such as *Medicago truncatula*, the mechanism and genetic regulation of polymerisation of the flavan-3-ols to PAs is still not fully understood [30, 32]. Furthermore, PAs in the above model species are produced primarily in the seed coat [32, 33], and not, as in sainfoin, in vegetative tissue. In sainfoin 12 cDNAs encoding genes involved in the flavonoid biosynthesis pathway were cloned and sequenced [34]. A better understanding of the regulation of PA accumulation in vegetative tissue is needed to facilitate breeding of sainfoin with improved PA content benefitting ruminant nutrition. Here we take a step in this direction by reporting the first annotated transcriptome library from sainfoin. It was used to identify genes involved in the PA biosynthesis pathways. We also provide data to demonstrate the potential for mining the transcriptome for simple sequence repeats (SSRs) and single nucleotide polymorphisms (SNPs).

## Methods

### Plant materials

We selected a set of five accessions representing a range of diversity [25–27]. The accessions are listed in Table 1. Seeds were germinated in standard potting compost M2 under controlled glasshouse conditions under a long-day photoperiod conditions (16/8 h light/dark). Seven day old whole seedlings of each sainfoin accession were collected and used for RNA extraction.

**Table 1:** *Onobrychis viciifolia* accessions selected for sequencing

Accession	Variety	Source
1363	Commercial sainfoin 1	Robert Salmon (Farmer)
1230	Visnovsky	GRIN
1005	Perly	RAU
1361	Zeus	Cotswold-Seeds Ltd
1364	Commercial sainfoin 2	Cotswold-Seeds Ltd

*GRIN* germplasm resources information network, *RAU* Royal Agricultural University, Cirencester

### RNA extraction

Total RNA were extracted from each accession from shoot and root tissue using the NORGEN Biotek Plant/Fungi with minor modifications (Norgen Biotek Corp, Ontario, Canada). The RNA was solubilized in 50 μl DEPC-treated H_2_O. Aliquots of 1.5 μl were taken for evaluation from each sample by gel electrophoresis and spectrophotometric analysis, respectively. The remaining RNA samples were immediately frozen at −80 °C. RNA was resolved on 1.0 % agarose gel stained with GelRed reagent (Biotium) and visualized by UV transillumination. The quantification was conducted using a spectrophotometer and Gen5 version 2.00.18 specified by the manufacturer using 1.5 μl of every RNA sample.

Once the quality of the RNA was validated, five pooled samples were generated. These consisted of 2 μl from root and 2 μl from aboveground extracts of the same plant, then diluted to 50 μl using nuclease-free, ultra-pure water. The pooled samples were used in cDNA library construction.

### cDNA library construction and illumina sequencing

The cDNA library was constructed following the protocol for TruSeq® RNA sample preparation v2 Guide Part # 15026495 Rev. F March 2014. This included library preparation, clustering and sequencing reagents. Briefly, the poly-A tail containing mRNA was purified using oligo-dT chains attached to magnetic beads, followed by washing steps to remove other RNA and any genomic DNA. After the purification, the mRNA was sheared in small fragments using divalent cations under high temperature. These RNA fragments were copied into first strand of cDNA using random primers and reverse transcriptase. After that, the second strand of cDNA was synthetized using DNA Polymerase I and RNase H. These final cDNA fragments then went through an end repair process where the addition of a single ‘A’ base takes place and ultimately the ligation of the adapters. The output was then purified and enriched using PCR to create the final cDNA library. The evaluation of the library was done by gel electrophoresis and UV transillumination.

The five cDNA libraries were sequenced with a HiSeq 2000 Desktop Sequencer from Illumina Sequencing Technologies. Paired-end (PE) reads were generated for the five cDNA libraries. Sequencing was optimized to generate 100 bp reads. All sequencing reads were deposited into the Short Read Archive (SRA) of the National Centre for Biotechnology Information (NCBI), and can be accessed under the Bioproject number PRJNA315368.

#### De novo assembly of transcriptome

The quality control of the raw reads and trimmed reads, as well as the study of the GC content of the sequences were performed with FastQC (http://www.bioinformatics.babraham.ac.uk/projects/fastqc/), version 0.10.1. [35].

Over-represented sequences are more common to the 3’ –end of the cDNA reads due to the substitution of ambiguous bases [36]. Further trimming and quality control of the reads was performed with the Trimmomatic programme [37]. The setting search parameters for the trimming were: removal of low quality sequence (limit = 0.05), removal of ambiguous nucleotides (maximal 2 nucleotides allowed) and removal of short sequences (minimum length: 50 nucleotides).

Trinity (version trinity/2013-02-25) (http://trinityrnaseq.sourceforge.net) was used to generate the combined assembly from all the reads as well as assemblies of the individual accessions [11]. All the reads from all genotypes were used to generate one combined assembly with Trinity. This combined assembly had a total of 215,219 contigs and was called Sainfoin Transcriptome 0.0. To reduce redundancy an in-house python script was used to keep only the longest isoform for each gene. This reduced the total amount of contigs (transcripts) to 92,772 and was called Sainfoin Transcriptome 1.0.

The assemblies were assessed for potential contamination [12]. The EST-Trimmer tool [38] was used to remove ambiguous sequences (any base call that was not an A, G, C or T), distal oligoN series and to develop a size cut-off. The minimum accepted size was 100 bp.

### Functional annotation and analysis

Putative genes were assigned to the global assembly using Blast2GO with BlastX against the refseq_protein database which was downloaded from NCBI on Jun 27th 2015 [13, 39]. The *e* value cut off was set at 10^−6^. Gene ontology terms (GO), pathway analyses using KEGG, and related statistics were identified. From KEGG a collection of genes encoding enzymes involved in the phenylpropanoid and PA biosynthesis were identified. BLAST [40] was used to compare this database with the Sainfoin Transcriptome 1.0 (*e* ≤ 10^−6^). The transcripts with hits against the PA biosynthesis gene database were extracted.

An expression analysis study of the Sainfoin Transcriptome 1.0 was performed using the Trinity RSEM functionality, and the results were visualised using MapMan (http://mapman.gabipd.org/web/guest/mapman) [41]. The main target during this step was to analyse the transcriptional level of the genes of particular interest, such as those involved in PA biosynthesis. Functional predictions were generated with the Mercator tool (http://mapman.gabipd.org/web/guest/app/mercator) [42] and MapMan was used to visualise the output in metabolic pathways [41]. We used the Wilcoxon Rank test to analyse which transcripts were most highly transcribed.

#### Phylogenetic analysis

Sainfoin Transcriptome 1.0 was compared with those of five other legume species and *Arabidopsis thaliana.* The analysis was performed as described previously [43]. Briefly, genes within pathways were compared with RAxML 8.0.22 [44] (100 bootstrap replications). The proteomes of six Fabaceae species and *A. thaliana* were aligned, and single gene clusters filtered and concatenated after removing gaps using HAL [45]. A phylogenetic tree based on these data was built with MEGA6 [46] using Maximum-likelihood and 100 bootstrap replications.

### SSR and SNP mining

The Perl script MISA (MIcroSAtellite; http://pgrc.ipk-gatersleben.de/misa/) [38] was used to identify SSRs in Sainfoin Transcriptome 1.0. The minimum number of nucleotide repeats searched during the SSR analysis was eight for di-nucleotide repeats, six for tri-nucleotides and five for tetra-, penta- and hexa-nucleotide repeats, respectively. For complex SSRs the maximum interval allowed between two sets of repeats was 50 bp. Oligonucleotides for amplifying SSRs were designed using BatchPrimer3 [47].

CLC Genomics Workbench v6.5 was used to identify SNPs. This software was used to align the reads from individual accessions using the Sainfoin Transcriptome 1.0. Initially, five lists were produced, one for each accession against the reference. This was filtered using the following thresholds: a minimum coverage of 20 and a maximum of 150. The upper threshold was set to minimise alignments to repetitive sequences. After this step further filtering was conducted to remove transcripts with more than five SNPs. The resulting SNPs were then merged into one file containing all the filtered SNPs from each accession, after removal of duplicate hits. Validation of SNPs was done by amplicon sequencing. PCR fragments from contigs were purified using MicroClean (Microzone Ltd, UK). The cleaned fragments were sequenced by capillary sequencing using an ABI 3730xl instrument (Life Sciences, Warrington, UK).

## Results

### Transcriptome assembly

In order to obtain a wide representation of the sainfoin transcriptomes, libraries from five different accessions were generated for Illumina sequencing. Sequencing of the libraries produced nearly 340 million 100-base raw paired end (PE) reads (Table 2). The overall G/C content was 43 %.

**Table 2:** Summary of the results obtained for each sainfoin cDNA library sequenced

Accession	Number of reads (paired)	Number of reads after trim	Percentage after trim	Avg. length after trim
1005	61,670,096	60,810,538	98.61 %	99.1
1363	92,477,926	91,234,366	98.66 %	99.3
1230	65,159,606	64,236,942	98.58 %	99.1
1364	49,407,102	48,563,967	98.29 %	98.2
1361	72,238,824	71,150,476	98.49 %	99.4
Total	340,953,554	335,996,289	98.53 %	99.0

Results show the number of reads that were recorded and their average length, the number of reads left after trimming (with Trimmomatic), their average length (Avg) and the percentage left after trimming

Quality control procedures resulted in a reduction of the total number of reads from 340 to 336 million reads (Table 2, Additional file 1). The remaining high-quality reads were used for the development of the *de-novo* transcriptome assembly of sainfoin.

The basic statistics of the libraries are summarised in Table 3. For Sainfoin Transcriptome 1.0 the N50 was 1224 and the mean length was 709. The longest transcript was 15,717 bp. Sainfoin Transcriptome 1.0 (Additional file 2) was then used as a reference for SNP and SSR identification, functional annotation and other downstream analyses.

**Table 3:** Statistics obtained from the five individual accessions and the combined assembly Sainfoin Transcriptome 1.0

Library	Total number of transcripts	N50	Mean length of transcripts	Longest transcript
Sainfoin Transcriptome 1.0	92,772	1224	709	15,717
1005	57,921	1449	776	15,641
1363	58,144	1484	794	15,492
1230	52,536	1494	804	15,745
1364	49,350	1493	806	15,595
1361	55,270	1481	795	16,953

### Functional annotation and analysis

**Fig. 1:**
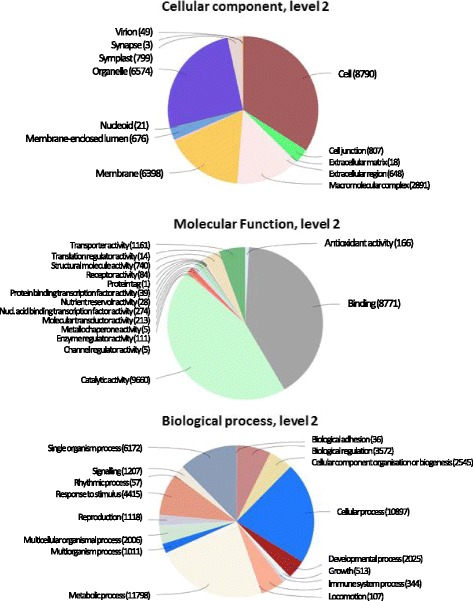
Gene ontology analysis of the *Onobrychis viciifolia* assembly. The pie charts show the percentage distribution of each GO type

Of the 92,772 transcripts processed with Blast2GO a little over 35200 produced BLAST hits. Approximately 16200 were not annotated and nearly 18,000 tags were assigned at least one GO annotation (Additional file 3). The highest number of GO annotations associated with one transcript was 15. In the Cellular Component category (Fig. 1), it can be seen that the main bulk of genes were related to the cell, organelles and membrane. In the Molecular Function category a high proportion of genes were involved in “catalytic activity”. This is likely to reflect the fact that at this young stage of development the seedling is devoting many resources to catabolism of reserves in the cotyledons and subsequent reallocation for growth of the shoot and root system. In the Biological Function class the three main categories were metabolic process, cellular process and single-organism process, also likely to be related to the fact that the seedlings were developing an intense metabolic activity to promote growth and establishment of the photosynthetic apparatus.

**Fig. 2:**
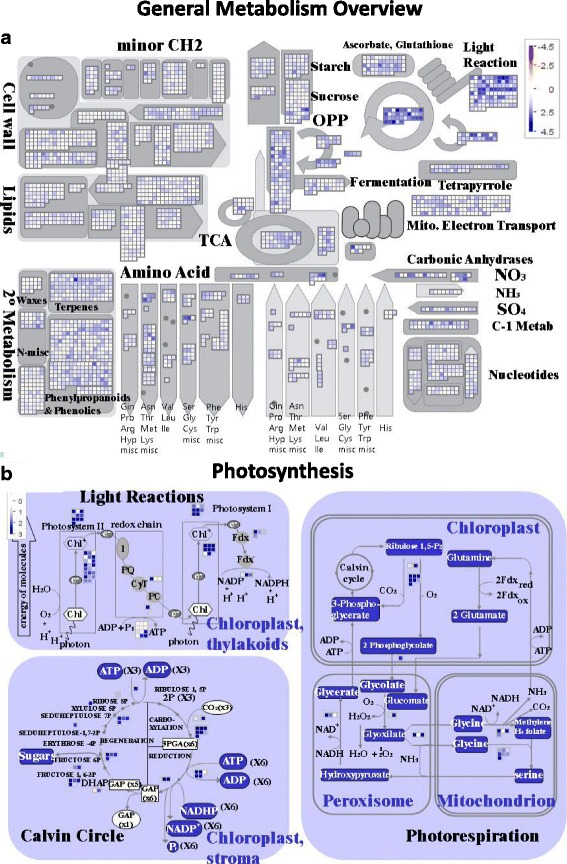
Association of the annotated genes of sainfoin to the general metabolism pathways (**a**) and the photosynthesis pathway (**b**)

The expression levels of each transcript are available in Additional file 4. Figure 2a gives an overview of which of the main metabolic pathways are most active in terms of gene expression. Figure 2b shows that genes involved in the photosynthetic light reaction complex and the Calvin cycle were highly active. To a lesser extent the main glycolytic pathway and the Citric acid cycle were also among the ones with more highly expressed genes. The RNA was extracted from very young plants (7 day old seedlings), so it is expected that genes involved in the establishment of the photosynthetic machinery are highly active, as well as glycolysis, gluconeogenesis, fatty acid, starch and sucrose metabolism. At that early stage of development reserves from the cotyledons are being mobilised to support growth and photosynthesis.

**Fig. 3:**
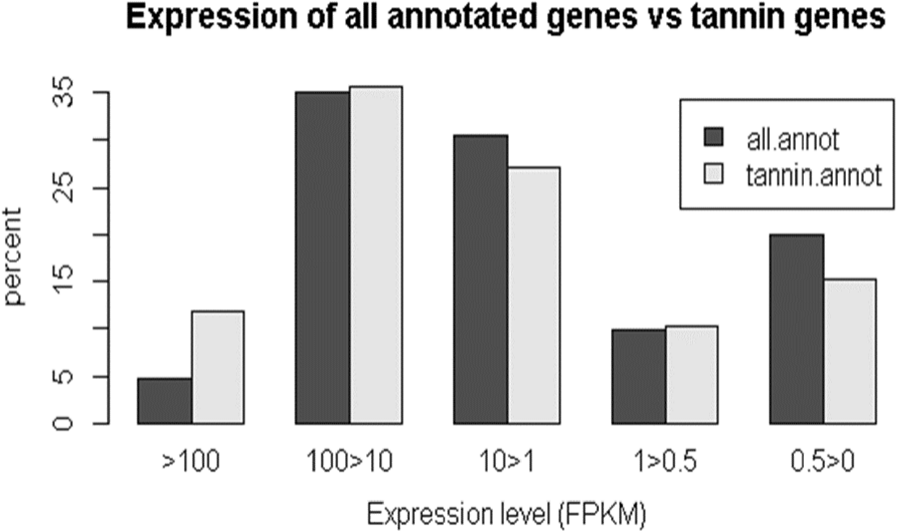
Comparison of transcription levels for the 63 genes annotated as involved in the biosynthesis of condensed tannins with those of all 18000 annotated genes

**Fig. 4:**
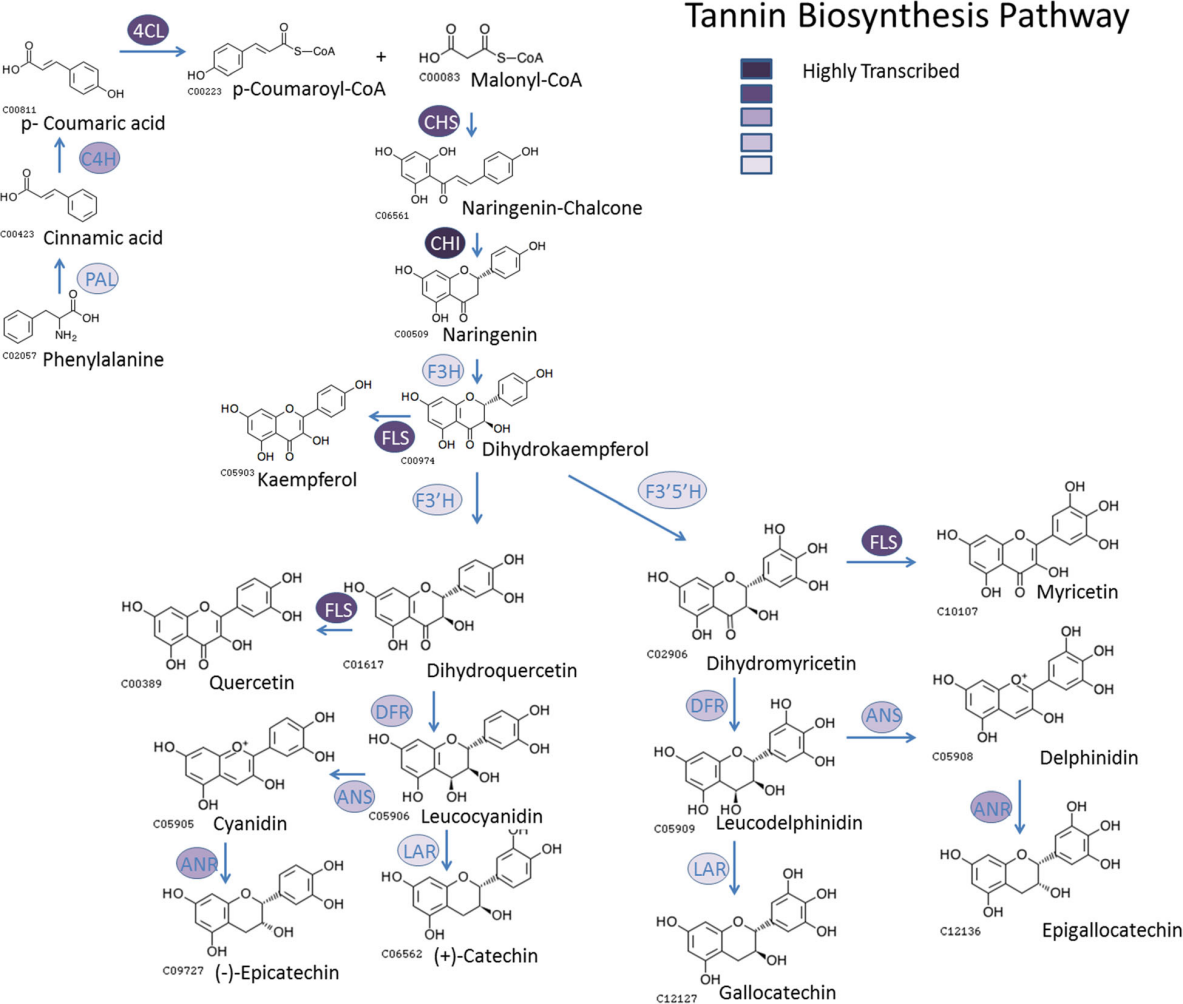
Tannin biosynthesis pathway representing the identified genes and relative transcriptional level. Note: Compounds have been adopted from KEGG and the proposed pathway has been modified from [61]

Of particular interest as far as sainfoin is concerned is the phenylpropanoid pathway leading to the biosynthesis of PAs. Figure 2a also indicates that this pathway is active, and this is illustrated in more detail in Figs. 3 and 4. We identified 63 contigs annotated as involved in the PA biosynthesis pathway (highlighted in green in Additional file 3). A closer look at those 63 contigs shows that the percentage of those expressed at high level (FPKM >100) was twice that of all the 18000 annotated genes. The frequency distribution at the other levels of expression was similar between the two groups (Fig. 3). A note of caution is warranted since the comparison is based on only 63 PA biosynthesis transcripts vs 18,000 genes annotated in total. Nevertheless, it would appear that even at this early stage of development some tannin genes are more highly expressed compared to the overall activity of the plant.

**Fig. 5:**
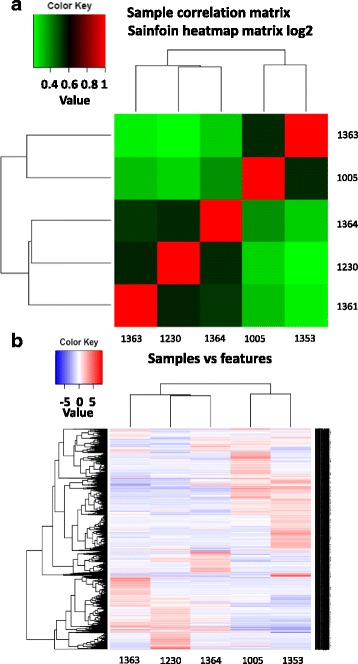
**a** and **b** Heatmap matrix showing relationship between the accessions used in the library construction in terms of expression levels of transcripts. Accessions: 1363 (commercial sainfoin 1, CS1), 1005 (Perly), 1364 (commercial sainfoin 2, CS2), 1230 (Visnovsky) and 1361 (Zeus)

The heatmaps of expression levels were obtained from the correlation of the five accessions (Fig. 5a) show that expression patterns of Perly (accession 1005) and “Commercial Sainfoin 1” (accession 1363) were most closely related. It may indicate genetical relatedness between the two. Zeus (accession 1361) and Visnovsky (accession 1230) and “Commercial Sainfoin 2” (accession 1364) formed another cluster. The first cluster is formed by two varieties which are typically considered as ‘common sainfoin’ while the second cluster is formed by varieties which are much taller and with higher yields, traditionally considered in between ‘common sainfoin’ and ‘giant sainfoin’. The heatmap samples versus features (Fig. 5b) confirm in more details for each transcript the previously observed correlation between accessions.

The ability of sainfoin to fix atmospheric nitrogen in symbiosis with rhizobium bacteria from the soil is another agronomically advantageous property of this legume. This process takes place in nodules formed on the roots of the plant. A lot is known at the molecular level about nodule formation and development, including the host plant genes involved in the signalling process with rhizobia [48]. Since RNA from roots of 7 day old seedlings was used in this work, it seemed appropriate to look for expression of genes involved in the early stages of nodule initiation and signal recognition of Nod factor produced by compatible rhizobia. This early stage of development is likely to be the time at which initial nodule formation takes place. Through the annotation done via Blast2Go, and searching the transcriptome with known nodulation signalling genes, we identified 17 genes. They are listed in Table 4 together with a summary of their annotation and the range of expression levels. It is interesting to note that two of the most highly expressed genes in the list are NFR1, and SYMRK. These are among the first genes involved in the interaction with rhizobia, as they are involved in nod factor binding and signalling response [48]. The NSP1 and NSP2 genes are involved further downstream in the nodulation process, and they are expressed at low levels. DMI1 is also downstream of the initial nod factor recognition, but upstream of the NSP genes in terms of the infection process, and was expressed at high levels.

**Table 4:** List of transcript tags from the sainfoin library that were identified as involved in nodulation by rhizobia

Transcript_ID	Annotation	Average expression	Expression range
comp91212_c0_seq1	Nodulation protein h-like	5.1	3.2–7.0
comp1255675_c0_seq1	Nodulation-signalling pathway 2 (NSP2)	0.3	0–0.5
comp101951_c0_seq2	Nodulation-signalling pathway 2 (NSP2)	4.28	1.1–8.2
comp97736_c0_seq1	Chitin elicitor receptor kinase 1-like isoform x1 (NFR1)	17.6	14.9–22.7
comp1757272_c0_seq1	Nodulation-signalling pathway 2 (NSP2)	0.4	0–2.0
comp50519_c0_seq2	Nodulation-signalling pathway 2 (NSP2)	0.4	0.3–1.0
comp99240_c2_seq1	Nodulation receptor kinase	7.1	5.3–9.5
comp100615_c0_seq2	Nodulation-signalling pathway 1 (NSP1)	3.0	1.2–4.7
comp82109_c0_seq2	Nodulation-signalling pathway 2 (NSP2)	0.6	0.3–1.0
comp97623_c0_seq1	Nodulation receptor kinase-like (SYMRK)	43.3	31.7–60.3
comp101444_c0_seq10	Nodulation protein h	5.7	3.7–7.2
comp95861_c0_seq1	Protein lyk5-like (NFR1)	3.2	0.7–6.7
comp2759407_c0_seq1	Nodulation-signalling pathway 2 (NSP2)	0.3	0–0.8
comp96879_c1_seq7	Chitin elicitor receptor kinase 1-like (NFR1)	2.4	0.6–4.2
comp98436_c0_seq3	DMI1	13.8	10.8–18.4
comp91752_c0_seq3	DMI1	18.6	13.9–25.3
comp71670_c0_seq1	NIN	0.6	0.2–1.3

The expression levels indicate the average and range between the five accessions that were used

*NFR1* nod factor receptor, *SYMRK* symbiotic receptor kinase, *DMI1* does not make infections 1, *NIN* nodule inception

### Phylogenetic analysis

**Fig. 6:**
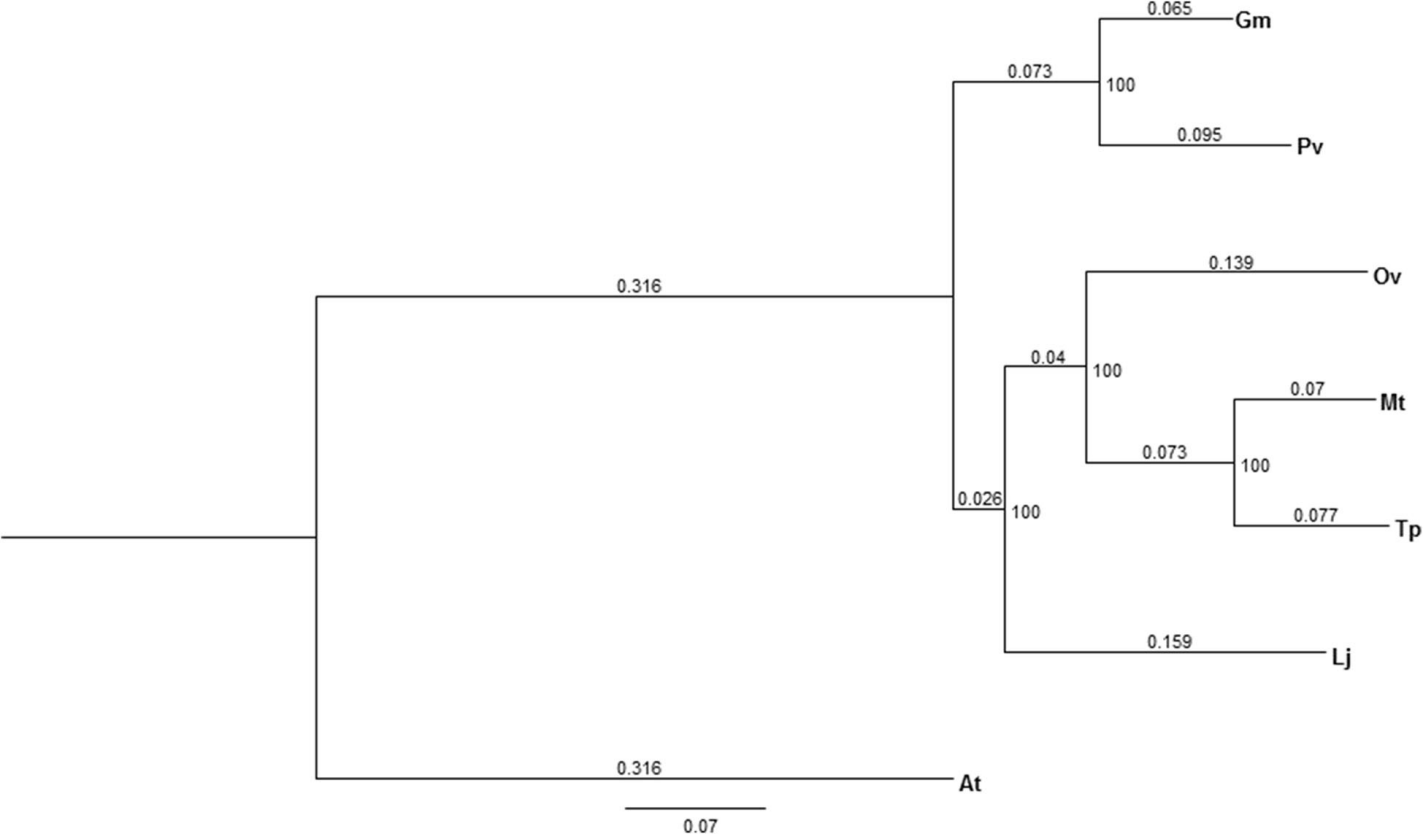
Phylogenetic tree illustrating the divergence of sainfoin from red clover and *M. truncatula* as well as other legumes with genomic information available. Gm (soybean); Pv (common bean); Ov (sainfoin); Mt (*M. truncatula*); Tp (red clover); Lj (*Lotus japonicus*); At (*Arabidopsis thaliana*). The latter is an outgroup. The numbers at the branchpoints are bootstrap values (based on 100 replications), and the values along the branches represent distances between clusters

The phylogenetic tree was based on the alignment of protein sequences of approximately 50 single copy number clusters present in *Arabidopsis thaliana* and the six Fabaceae species, soybean (Gm), common bean (Pv), *Lotus japonicus* (Lj), *Medicago truncatula* (Mt), sainfoin (Ov) and red clover (Tp). The tree is shown in Fig. 6. Sainfoin, *M. truncatula* and red clover form a cluster of three closely related species, while *L. japonicus* and particularly soybean and common bean are more distantly related to sainfoin.

### Detection of polymorphisms

Using the MISA tool we identified 3823 SSRs in 3575 sequences in Sainfoin Transcriptome 1.0. The frequency distribution of the microsatellites with di-, tri-, tetra-, penta- and hexa-nucleotide repeats were 39.73 %, 44.13 %, 15.62 %, 0.42 % and 0, respectively. The identified SSRs are provided in Additional file 5. Over 100 SSRs from this list were validated in a study of sainfoin germplasm diversity [49].

We also found six potential SSRs in transcripts involved in the PA biosynthesis pathway. They were identified in genes coding for: i) chalcone isomerase, ii) isoflavone 2’-hydroxylase-like; iii) flavonol 3-O-methyltransferase, iv) coumarate 4-hydroxylase; v) flavonoid3-O-glucosyltransferase; vi) anthocyanidin synthase (Additional file 5).

Detection of SNPs: the initial files from each accession contained about 500 K SNPs; after filtering by coverage there were approximately 120–135 K SNP variants. After further filtering to remove transcripts with more than five SNPs the number of SNPs in each file was reduced to approximately 12–15 K. They were then merged into one file containing all the filtered transcripts from each accession. This resulted in a final number of approximately 77 K SNPs (Additional file 6). For validation we used DNA isolated from four sainfoin genotypes representing the accessions Cotswold Common, Perly, Visnovski and Zeus. We were able to amplify and sequence four contigs annotated as involved in the flavonoid biosynthesis pathway and containing 12 putative SNPs. Ten of those were confirmed in at least two of the four genotypes. The results are summarised in Additional file 7.

## Discussion

To our knowledge, this is the first *de novo* transcriptome assembly described for sainfoin, and thus provides the first large scale molecular resource for future genetic studies and breeding programmes.

In this experiment we used young seedlings from five different accessions. We experienced problems with extracting sufficient RNA of high quality from older plant material. It seemed reasonable to assume that PAs and other secondary metabolites were a likely cause. The choice of young seedlings was made to minimise this issue [50, 51]. On the other hand, it was important to capture the genes involved in this important secondary metabolism pathway, as PAs are considered responsible for the beneficial attributes of sainfoin such as, for example, its resistance to many common pests and diseases, antiparasitic activity, in vitro methane reduction and prevention of bloat [1–5, 52–54]. This work showed that the genes of the phenylpropanoid pathway are transcribed at this early stage of the plants development (Fig. 4). We were able to identify 63 transcripts from this pathway including those producing anthocyanin and leucoanthocyanidin. In addition, we were able to identify homologues of the transcription factor gene *TT8*, the transport factor *MATE* or *TT12*, and the proton pump *AHA10*. The former has been implicated in regulating the expression of *TT12* and *AHA10* [31, 55]. We also identified a gene with strong homology to a laccase gene in *A. thaliana* suspected of involvement in the polymerisation of flavonols to proanthocyanidins [56]. In total, we identified 23 transcripts of these four genes. They are highlighted in blue in Additional file 3. More detailed analyses with different type of tissues such as leaf material of plants at different stages of growth, different varieties or plants cultivated in different environments should be considered. Our priority here was to develop a functional genomics resource of the transcribed part of the genome. Secondly, we wanted to use the resource to identify molecular markers, in particular SSRs and SNPs in genic regions.

The total size of the Sainfoin Transcriptome 1.0 was nearly 67 Mb. The genome size of sainfoin has been estimated to be 1223 Mbp or 2.5 pg [25]. Sainfoin Transcriptome 1.0 thus represents 5.5 % of the total genome. The heterozygous condition of sainfoin, and its potential allogamous habit [57], may have resulted in some duplicate or allelic transcripts. Therefore, the suggested figure above could be an overestimate. Nevertheless, this research represents a significant advance in sainfoin functional genomics resources. The median contig length (N50) obtained in Sainfoin Transcriptome 1.0 was 1224 and the mean length was 709. The combined assembly had the largest number of transcripts, which were 92,772, followed by accession 1363 which had 59,144 transcripts (Table 3). However, Sainfoin Transcriptome 1.0 had a smaller N50 and mean length than the individual assemblies. This is due to the fact that the combined assembly is larger so has a longer tail of smaller transcripts, which skews the mean length and the N50 value. This is supported by comparing the assembly statistics for the combined vs the individual assemblies with increasing minimum length threshold values. With minimum length thresholds of 300, 700, 1500 and 10000, the N50 values for Sainfoin Transcriptome 1.0 were 1474, 1970, 2554 and 12556, respectively. For the individual assembly of Perly (accession 1005) the N50 values were 1655, 2027, 2524 and 12263, respectively. This shows that when the cut-off size is at 1500 or above, the N50 for the combined assembly is larger than for the Perly assembly, thus confirming our interpretation. The trends were similar for all five individual assemblies.

The phylogenetic analysis performed here show that sainfoin is closely related to red clover as expected. However, it is equally closely related to *M. truncatula* (Fig. 6). Our expectation was that red clover and sainfoin would be closest in terms of phylogeny as they both have the same basic number of 7 chromosomes. The fact that they are not any closer to each other than to *M. truncatula* would suggest that the chromosome rearrangements and reductions in sainfoin and red clover have occurred independently. Genome duplication events and tetraploidy are also likely to have contributed to the divergence from red clover and *M. truncatula.* To what extent synteny is conserved between sainfoin and other legumes remains to be seen. To answer this question would require a genome assembly and/or dense genetic maps.

Nearly 18,000 GOs were annotated in Sainfoin Transcriptome 1.0, representing a potentially useful platform for future candidate gene examination. The annotation, analysis and ontology showed that the results obtained for cellular components, molecular functions and biological processes were similar to those obtained from related species such as *Trifolium pratense* (red clover) [58] (Figs. 1 and 2). Some pathways significantly expressed were the citrate cycle, glycolysis/glyconeogenesis pathway, fatty acid degradation and amino acid metabolism (Fig. 2). This is likely to reflect the fact that the resources of storage protein and energy in cotyledons are being remobilised to develop new tissues such as for example stems and leaves. Indeed, the carbohydrate metabolism responsible for the development of new tissues was highly transcribed (Fig. 2). The photosynthesis pathway was also very active, indicating that the photosynthesis apparatus of these young seedlings has been established and is capable of providing the necessary energy to synthetize carbohydrates to build new tissues and sustain further growth. The energy obtained from the photosynthesis is also important for two key processes in sainfoin, nitrogen fixation and transpiration. The cost of these processes are likely to be partly responsible for sainfoin low yields and heat tolerance [9]. The high transpiration rate is most likely related to the long tap root system which gives sainfoin access to more water in the soil than most other legumes and forage crops [25]. A modern plant breeding programme to improve sainfoin yields, should consider optimising the energy metabolism of the plants, especially during the first stages. As with many legumes slow establishment can be linked to the development phase of nitrogen fixing root nodules, during which time it is at a disadvantage compared to non-legume companions such as forage grasses [59]. Sainfoin root cDNA were included in this study. This is the stage at which nodule initiation and development is taking place. The Blast2GO annotation identified 17 genes, homologues of which are known to be involved in the very beginning of the nodule initiation and signalling processes (Table 4). Consistent with their role in nod factor recognition and signalling, the initial interaction with rhizobia, NFR1 and SYMRK were expressed at high levels. The NSP1 and NSP2 genes are involved further downstream in the nodulation process, and they were expressed at lower levels, which could mean that these signalling processes are not yet fully active. The selection of sainfoin variates capable of developing a faster and more efficient nodulation could indirectly promote the growth of the plant, and thus reduce the competition problem. A more robust and early developed canopy of sainfoin is necessary to prevent field invasion by weeds, and would have a positive impact on yield in the first year.

The assembly, allowed us to identify 3786 SSRs. To use next generation sequencing techniques to identify SSRs is a very cost-effective technique. Over 100 of the SSRs identified here have been validated and used in a genetic diversity study of sainfoin germplasm [50]. The identification of SSRs in a *de novo* assembly was easier to address than the development of SNPs, which is challenging without a reference genome. The combined assembly was used to align the raw reads. The complex genetics of sainfoin in terms of tetraploidy, uncertainty about whether it is allo- or auto-tetraploid [25] is likely to compound the challenge. To minimise the influence of these factors, we set quite stringent thresholds for coverage in order to reduce the risk of identifying sequence errors rather than SNPs, and on the other hand to avoid too many hits to repetitive sequences. The potential value of the SNPs identified here (Additional file 6) will only be proven by experimental validation. However, to attain some indication of their validity we utilized a previously published [34] set of 12 transcripts. Using BLAST we aligned this data set against our transcriptome library, and compared the SNPs found in these transcripts with our own. In 2 of the 12 transcripts we found identical SNPs. For the other ten transcripts, some putative SNPs had been filtered from our SNP list, or else there were no SNPs to compare. We validated experimentally ten SNPs of a total of 12 from a small subset of contigs annotated as encoding genes involved in the flavonoid biosynthesis pathway (Additional file 7). We did not have access to DNA from the same plants that were used for RNA extraction, so the genotypes used for validation were genetically distinct from those providing the RNA, thus introducing potential ascertainment bias. The other issue with polymorphisms in sainfoin relates to its tetraploidy. Methods for distinguishing homoeologous from allelic SNPs have been described, but such methods are usually dependent upon access to a reference genome [60].

## Conclusions

The present study represents the first comprehensive RNA-Seq approach in the non-model species sainfoin to generate functional genomic resources for modern molecular breeding approaches to improve this tannin containing forage crop. The *de novo* study of a tetraploid species involves some challenges due to inherent difficulties in distinguishing true homoeologues and paralogues from duplications and assembly artefacts. In order to overcome these issues we designed an experimental procedure where a combined assembly was developed using pooled reads samples. This improved the coverage and depth of sequencing. This study has provided information about genetic diversity and a phylogenetic analysis of sainfoin, a large set of putative SNPs, SSRs and candidate genes directly from sainfoin, providing valuable resources for future genetic studies.

## Additional files

Additional file 1: Summary of trimming of sequencing reads. Table with summary of the trimming of the sequencing reads. (DOCX 12 kb) Additional file 2: Transcriptomics assembly. FASTA file of 92772 sainfoin transcripts. (ZIP 26489 kb) Additional file 3: Annotation of the sainfoin assembly. Excel file with list of annotated contigs from Sainfoin Transcriptome 1.0. Contig names in green indicate transcripts encoding genes involved in the flavonoid biosynthesis pathway. Contigs in blue indicate transcripts with potential involvement in proanthocyanidin biosynthesis. (XLSX 1685 kb) Additional file 4: Expression levels (FPKM) of the transcripts of the sainfoin assembly. Excel file with expression levels (FPKM) of transcripts from each of the five sainfoin accessions. (XLSX 4336 kb) Additional file 5: SSRs identified in the sainfoin combined assembly. Excel file with list of SSR motifs identified in transcripts from Sainfoin Transcriptome 1.0. (XLSX 498 kb) Additional file 6: SNP polymorphisms in the five sainfoin transcript assemblies. A csv file listing putative SNP variants identified in one or more of the five sainfoin transcriptome assemblies. (CSV 6315 kb) Additional file 7: Experimentally validated SNPs. Excel file summarising the experimentally validated SNPs. (XLSX 11 kb)

## Abbreviations

BLAST: Best local alignment search tool
EST-SSR: Expressed sequence tag-simple sequence repeats
FPKM: Fragments per kilobase per million
GO: Gene ontology
KEGG: Kyoto encyclopedia of genes and genomes
PA: Proanthocyanidins
