# Supplementary material for: De-novo transcriptome assembly for gene identification, analysis, annotation, and molecular marker discovery in Onobrychis viciifolia
Source: BMC Genomics. 2016 Sep 26;17:756. doi: 10.1186/s12864-016-3083-6 (PMC5037894; doi:10.1186/s12864-016-3083-6)
Supplement: Additional file 1: — Summary of trimming of sequencing reads. Table with summary of the trimming of the sequencing reads. (DOCX 12 kb) [file 12864_2016_3083_MOESM1_ESM.docx]

Additional File 1.

Table S1. Detailed trim results after the Trimmomatic analysis summarising the number of reads that were processed, reads that did not need to be trimmed, reads that needed to be trimmed and number of them that were trimmed or discarded.

| **Trim** | **Input reads** | **No trim** | **Trimmed** | **Discarded** |
| --- | --- | --- | --- | --- |
| Trim on quality | 340,953,554 | 320,740,104 | 19,391,888 | 821,562 |
| Ambiguity trim | 340,131,992 | 339,632,425 | 469,608 | 29,959 |
| Filter on length | 340,102,033 | 335,996,289 | 0 | 4,105,744 |
